# Meteorological Observations

**Published:** 1860

**Authors:** J. G. Westmoreland

**Affiliations:** Smithsonian Institution, Jan., 1860, at Atlanta, Ga.


### METEOROLOGICAL OBSERVATIONS,

Taken by J. G. Westmoreland, M. D., for the Smithsonian Institution, Jan., 1860, at Atlanta, Ga., Latitude 33 deg. 45 min.—Longitude 7 deg. 30 min..—Height above the Sea, 1050 feet.

| Days of Mo | BAROMETER. | | | THERMOMETER. | | | RAIN AND SNOW. | | CLOUDS. | | | WIND. | | |
|---|---|---|---|---|---|---|---|---|---|---|---|---|---|---|
| | 7 A. M. | 2 P. M. | 9 P. M. | 7 A. M. | 2 P. M. | 9 P. M. | amt.rn | in.sn'w | 7 A. M. | 2 P. M. | 9 P. M. | 7A.M. | 2P.M. | 9P.M. |
| 1 | 29.95 | 30.00 | 30.05 | 18 | 26 | 20 | | | 5 | 5 | 5 | N W | N W | N W |
| 2 | 30.10 | 30.20 | 30.20 | 10 | 26 | 18 | | | 0 | 0 | 0 | N W | N W | N W |
| 3 | 30.20 | 30.25 | 30.20 | 18 | 32 | 30 | | | 0 | 0 | 0 | N W | S W | W |
| 4 | 30.15 | 30.10 | 30.15 | 26 | 36 | 32 | | | 0 | 0 | 0 | W | W | W |
| 5 | 30.20 | 30.30 | 30.25 | 26 | 38 | 32 | | | 0 | 0 | 0 | N | N | N |
| 6 | 30.25 | 30.12 | 30.00 | 26 | 32 | 32 | | | 5 | 5 | 10 | N E | N E | E |
| 7 | 29.80 | 29.75 | 29.85 | 36 | 38 | 40 | 62 | | 10 | 10 | 10 | E | E | E |
| 8 | 29.95 | 30.00 | 30.00 | 42 | 56 | 38 | | | 5 | 0 | 0 | W | W | W |
| 9 | 30.00 | 30.10 | 30.10 | 38 | 52 | 48 | | | 5 | 0 | 0 | W | W | W |
| 10 | 30.10 | 30.10 | 30.05 | 36 | 46 | 44 | | | 5 | 10 | 10 | S W | S W | S W |
| 11 | 30.00 | 30.05 | 30.05 | 42 | 56 | 52 | | | 10 | 5 | 5 | S W | S W | S E |
| 12 | 30.10 | 30.15 | 30.10 | 52 | 60 | 48 | | | 10 | 10 | 10 | S | S | S W |
| 13 | 30.10 | 30.10 | 30.10 | 48 | 50 | 58 | 25 | | 10 | 10 | 10 | W | W | W |
| 14 | 29.90 | 29.95 | 29.90 | 48 | 52 | 50 | 25 | | 10 | 10 | 10 | S E | S E | S E |
| 15 | 29.85 | 29.90 | 29.80 | 40 | 50 | 40 | | | 5 | 5 | 5 | E | E | E |
| 16 | 29.85 | 29.85 | 29.80 | 32 | 40 | 32 | | | 0 | 0 | 0 | S | S W | N W |
| 17 | 29.76 | 29.85 | 29.82 | 32 | 42 | 32 | | | 5 | 0 | 5 | N W | N W | N |
| 18 | 29.82 | 29.85 | 29.73 | 32 | 48 | 43 | | | 5 | 0 | 0 | N W | S W | S W |
| 19 | 30.03 | 30.15 | 30.05 | 32 | 49 | 40 | | | 0 | 0 | 0 | S | N W | N W |
| 20 | 30.03 | 30.05 | 30.07 | 32 | 50 | 48 | | | 0 | 0 | 0 | S W | S | S |
| 21 | 30.07 | 30.10 | 30.05 | 38 | 58 | 50 | | | 0 | 0 | 0 | N W | S W | W |
| 22 | 30.00 | 30.10 | 30.10 | 40 | 60 | 50 | | | 0 | 0 | 0 | W | N W | N W |
| 23 | 30.12 | 30.22 | 30.20 | 40 | 58 | 48 | | | 0 | 0 | 0 | N | E | E |
| 24 | 30.20 | 30.30 | 30.20 | 42 | 60 | 50 | | | 0 | 0 | 0 | S E | S E | S E |
| 25 | 30.20 | 30.20 | 30.15 | 48 | 60 | 58 | | | 0 | 0 | 0 | S | S | S |
| 26 | 30.10 | 30.02 | 29.95 | 48 | 62 | 58 | | | 0 | 0 | 0 | S | S | S |
| 27 | 29.90 | 29.95 | 30.05 | 34 | 34 | 34 | | | 10 | 5 | 0 | N W | N W | N W |
| 28 | 30.00 | 30.05 | 30.05 | 30 | 50 | 36 | | | 0 | 0 | 0 | N W | N W | N W |
| 29 | 30.05 | 30.15 | 30.10 | 34 | 56 | 46 | | | 0 | 0 | 0 | N W | N W | N W |
| 30 | 30.05 | 30.15 | 30.10 | 42 | 60 | 50 | | | 5 | 5 | 5 | W | W | W |
| 31 | 30.00 | 29.85 | 29.75 | 46 | 52 | 54 | 75 | | 10 | 10 | 10 | S W | S W | S W |

Explanation of Table.—0 Signifies Entire Clearness—5 Half Cloudy—10 Entire Cloudiness.

We regret to learn that a fatal endemic of puerperal fever has appeared in Bellevue Hospital. There would seem to be indications of an atmospheric condition in and around New York thus early in the winter, which increases the hazard of parturition.—Cases of puerperal convulsions, as well as puerperal fever, are not unfrequent. Many of the former are due to uræmic poisoning of the blood, and are too often fatal. Erysipelas seems to be simultaneously prevailing, and there have been an unusual number of typhoid fever cases. Still, however, the mortality of the city has not attained any formidable magnitude.—*American Medical Gazette.*

